# Antimicrobial stewardship interventions to minimize healthcare worker exposure to SARS-CoV-2

**DOI:** 10.1017/ice.2020.302

**Published:** 2020-06-19

**Authors:** K. Ashley Jones, Matthew Watson, Jesse T. Jacob, Zanthia Wiley

**Affiliations:** 1 Department of Pharmaceutical Services, Emory Healthcare, Atlanta, Georgia; 2 Division of Infectious Diseases, Department of Medicine, Emory University School of Medicine, Atlanta, Georgia

*To the Editor*—Antimicrobial stewardship programs responded to the coronavirus disease 2019 (COVID-19) pandemic by developing treatment pathways to monitor the use of potential COVID-19 therapies to ensure appropriate management and to mitigate toxicities.^1,2^ These approaches mimicked traditional stewardship efforts by using the shortest effective duration of therapy, assessing drug–drug interactions, and monitoring drug safety and efficacy parameters. Given the rapid rise in the number of patients with confirmed or suspected COVID-19 cases combined with the national shortage of personal protective equipment (PPE), we identified an immediate opportunity for pharmacists, as integral members of the antimicrobial stewardship team, to help conserve PPE and limit healthcare worker (HCW) exposure by consolidating the number of times medications needed to be administered throughout the day.

In our hospital’s pharmacy practice model, decentralized pharmacists perform daily patient chart reviews to optimize pharmacotherapy, including antimicrobials. Pharmacists utilize a clinical surveillance software (Theradoc, Premier, Charlotte, NC) using both real-time alerts and structured workflows for anticoagulation monitoring, renal dose adjustments, therapeutic drug monitoring, and microbiology review. We leveraged existing work flow and infrastructure to develop this consolidation initiative. By incorporating positive SARS-CoV-2 PCR results into pharmacists’ daily workflow via our surveillance software, pharmacists can easily identify these patients and assess their medication administration record for optimization. Persons under investigation were identified during routine chart review.

The initiative provided pharmacists with guidance on strategic methods of consolidating the medication administration record based on 3 domains: (1) consolidation of medication administration times, (2) optimizing pharmacotherapy, and (3) therapeutic drug monitoring.

## Consolidation of medication administration times

Throughout the course of a patient’s hospital stay a patient’s medication administration records can become complex as medications are added and administration times are changed. For example, at our institution, if a prescriber orders a medication every 24 hours, the medication administration time will default to the next hour. If the start time is not critical, the medication could be given at a time when other medications are already scheduled. Additionally if a medication is ordered every 8 hours (default times: 6:00 am, 2:00 pm, and 10:00 pm), modifying the administration times to 9:00 am, 4:00 pm, and 9:00 pm would align better with medications ordered every 12 hours (default times: 9:00 am and 9:00 pm) and would reduce the number of times nurses would need to enter the room and use PPE. We provided guidance to pharmacists to consolidate medication times while considering safety of early or late doses during the transition and potential drug–drug interactions (eg, doxycycline with calcium supplementation). High-risk medications such as antimicrobials, antiepileptic agents, anticoagulants, and immunosuppressive agents required consultation with the provider.

## Optimizing pharmacotherapy

We encouraged pharmacists to recommend therapy modifications that maintained both safety and efficacy, while decreasing exposure to HCWs. For example, a patient on twice-daily isophane insulin as an outpatient may be able to switch to once-daily long-acting insulin as an inpatient. This approach later led to the implementation of a streamlined protocol for managing mild-to-moderate diabetic ketoacidosis with subcutaneous insulin. Pharmacists also focused on opportunities to switch patients from intravenous to oral therapy based on our hospital’s protocol as a means to reduce entering patient rooms, since oral therapy only requires 1 visit, but intravenous therapy requires a second visit after the infusion is complete. Furthermore, pharmacists advocated for stopping antibiotics in patients with confirmed SARS-CoV-2 but no microbiologic evidence of bacterial infection. Discontinuing unnecessary antimicrobials reduces the risk of adverse effects including *Clostridiodes difficile* infection, which would further complicate a patient’s hospital course and likely result in increased use of PPE for additional medication administration.

## Therapeutic drug monitoring

At our institution, pharmacists are responsible for vancomycin dosing and monitoring. We encouraged pharmacists to reduce unnecessary testing (eg, uncomplicated skin and soft-tissue infections or anticipated short course of therapy) to help further decrease HCW exposure. If the pharmacist determined that a test was needed, the pharmacist placed a timed order or a phlebotomy or nursing order. We partnered with phlebotomy leadership to determine their high-volume times for routine laboratory tests, and we reviewed their staffing model. Ideal times were identified in the morning and the evening for patients located in both the intensive care units and wards to collect samples for testing vancomycin levels and to minimize PPE use. Pharmacists used these preferred times to obtain samples to test levels or extrapolated levels if needed.

These efforts have reduced unnecessary patient room entry, minimized HCW exposure, and conserved PPE supply. Our interventions serve as a model for leveraging the collaborative relationship between pharmacists and antimicrobial stewardship programs during the COVID-19 pandemic. With some modifications to accommodate other institutions’ work flows, this initiative can be adapted by other antimicrobial stewardship programs and pharmacy departments. During these challenging times, it is imperative to engage in multidisciplinary collaboration to not only keep the patient safe but our own colleagues as well. We hope our project inspires other creative ways for antimicrobial stewardship programs to contribute to efforts to prevent HCW exposure to SARS-CoV-2.

## References

[1] Stevens MP , Patel PK , Nori P. Involving antimicrobial stewardship programs in COVID-19 response efforts: all hands on deck. Infect Cont Hosp Epidemiol 2020;41:744–745. 10.1017/ice.2020.69 PMC7137534 32167442

[2] Stevens RW , Estes L , Rivera C. Practical implementation of COVID-19 patient flags into an antimicrobial stewardship program’s prospective review. Infect Cont Hosp Epidemiol 2020;15:1–2. 10.1017/ice.2020.133 PMC7184145 32290883

